# How and why to use ‘vulnerability’: an interdisciplinary analysis of disease risk, indeterminacy and normality

**DOI:** 10.1136/medhum-2023-012683

**Published:** 2023-09-11

**Authors:** Andrea Ford, Giulia De Togni, Sonja Erikainen, Angela Marques Filipe, Martyn Pickersgill, Steve Sturdy, Julia Swallow, Ingrid Young

**Affiliations:** 1 Centre for Biomedicine Self and Society, The University of Edinburgh Usher Institute of Population Health Sciences and Informatics, Edinburgh, UK; 2 Department of Sociology, University of Aberdeen, Aberdeen, UK; 3 Department of Sociology, Durham University, Durham, UK; 4 Usher Institute for Population Health Sciences and Informatics, University of Edinburgh, Edinburgh, UK; 5 School of Social and Political Science, The University of Edinburgh College of Humanities and Social Science, Edinburgh, UK

**Keywords:** Ethics, COVID-19, Medical humanities, Philosophy, Social science

## Abstract

In recent years, ‘vulnerability’ has been getting more traction in theoretical, professional and popular spaces as an alternative or complement to the concept of risk. As a group of science and technology studies scholars with different disciplinary orientations yet a shared concern with biomedicine, self and society, we investigate how vulnerability has become a salient and even dominant idiom for discussing disease and disease risk. We argue that this is at least partly due to an inherent indeterminacy in what ‘vulnerability’ means and does, both within and across different discourses. Through a review of feminist and disability theory, and a discussion of how vulnerability and disease both get recruited into a binary conceptualisation of normal versus abnormal, we argue that vulnerability’s indeterminacy is, in fact, its strength, and that it should be used differently than risk. Using COVID-19 management in the UK as an illustration of the current ambivalence and ambiguity in how vulnerability versus risk is applied, we suggest that instead of being codified or quantified, as it has started to be in some biomedical and public health applications, vulnerability and its remedies should be determined in conjunction with affected communities and in ways that are polyvalent, flexible and nuanced. The concept of vulnerability encapsulates an important precept: we must recognise inequality as undesirable while not attempting to ‘solve’ it in deterministic ways. Rather than becoming fixed into labels, unidirectional causalities or top-down universalising metrics, vulnerability could be used to insist on relational, context-specific understandings of disease and disease risk—in line with contemporary social justice movements that require non-hierarchical and non-universal approaches to problems and solutions.

## Introduction

 In recent years the concept of vulnerability has been gaining traction in theoretical and professional spaces interested in medicine and health. Critical and activist scholarship has theorised health vulnerability in terms of disability, race and gender justice, and has drawn attention to underlying structural and epistemic conditions (eg, Chung 2021). Although vulnerability has been a key term in medical ethics and bioethics since at least the 1970s, there has been renewed attention and recent recognition that it should be further explored (Levine *et al*. 2004; Rogers, Mackenzie, and Dodds 2012). Vulnerability is now ubiquitous in global public health and applied development studies, often twinned with ‘resilience’, and various methods have been proposed to systematise and quantify it (eg, Bourgois *et al*. 2017; Luna 2019). It has also shown up as a technocratic term—for example, in government protocols for COVID-19 (Department of Health and Social Care 2022; Lancet 2020). In and across these spaces there is ambiguity and ambivalence about whether and how vulnerability should be driving decisions, and even what it means.

We find it useful to revisit what distinguishes vulnerability from a concept that has much in common with it; namely, risk. From Ulrich Beck’s influential *Risk Society* published in 1992 (original German 1986), to Lash, Wynne, and Szerszynski (1996), to Lupton (1999), to give only a few possible examples, social scientists have used the ways risk is embedded in bureaucratic and governmental apparatuses, as well as the conditions for basic citizenly and personal activities, to analyse contemporary life. Risk is ubiquitous as a guiding principle within medicine and public health, reflected by the turn-of-the-century establishment of the journal *Health, Risk and Society* and the increasing management of health through surveillance, pre-emptive pharmaceuticals and diagnostic testing.

Both risk and vulnerability are context dependent and historically situated in terms of how they are applied. However, while risk has long since become an actuarial science, a problem of statistics and mathematical models of uncertainty (although applied within sociocultural settings and markets), vulnerability retains far more of its qualitative and subjective dimensions. This can result in a ‘vagueness’ that is useful for strategically mobilising resources, attention and public concern to public health issues, yet that masks the power dynamics at work (Katz *et al*. 2020). Meanwhile, some political philosophy argues for a conception of ‘structural health vulnerability’ that foregrounds the power dynamics that produce inequalities and the resulting differential health risks (Chung 2021). Recent work has also asserted the need to consider how medical practice exacerbates experiences of vulnerability over and above its inherent and structural aspects (Coyle and Atkinson 2019). How to use ‘risk’ versus ‘vulnerability’ is being worked out in practice at this moment, and the apparent appeal of vulnerability is worth interrogating. We suggest that this appeal is at least partly due to an inherent indeterminacy in what ‘vulnerability’ means and does, both within and across different discourses. This indeterminacy allows the concept to be used in polyvalent, flexible and nuanced ways that might advance social justice, but also means that when its application is modelled off risk and approached deterministically, the usage becomes ambiguous, ambivalent and vague, and stymies justice.

## Position and approach

We, the authors, are writing as a group of scholars with different disciplinary orientations towards theoretical, professional and popular spaces. We are, though, all qualitative researchers, and engaged to different degrees with medical humanities and science and technology studies (STS). Within our collaborative discussions, we have noticed that vulnerability is an emerging way of situating and approaching disease, and arguably a key concept in the contemporary landscape of how disease is understood. We believe our interdisciplinary perspective can illuminate some of the context and stakes of this development, as we combine backgrounds in anthropology, bioethics, history, philosophy and sociology. To varying degrees, we draw from and participate in gender studies and feminist theory, critical public/global health, disability studies, and anticolonial and critical race theory.

Through coming together to discuss the valences of vulnerability, we grappled both with the different ways we had encountered the term as well as a range of orientations towards the methods and aims of scholarship. This includes scholarship’s normative dimensions; that is, to what extent and in what ways our scholarship promotes a vision of the world as it *should* be. Of course, to some extent any critical work promotes a normative view, and no description can be neutral. Still, some disciplinary traditions are more comfortable than others with producing openly prescriptive work. Our collaboration also reinforced a need to distinguish between actors’ categories and analysts’ categories—that is, when are *we* using the term vulnerability to describe something about the world, versus describing how ‘people in the world’ are using vulnerability to characterise bodies and agents, and to take actions. Through considering manifestations of vulnerability across theoretical, professional and popular spaces, we have sought to draw from the strengths of our multiple analytical and empirical approaches. Given the conceptual nature of this paper, it was not appropriate or possible to involve patients or the public in the design, or conduct, or reporting, or dissemination plans of our research.

In terms of how vulnerability fits into the critical and sociocultural study of medicine, for instance, some scholars orient towards ‘vulnerability’ as an analytical construct in a similar way to feminist and disability theory, while for others it is an empirical object. Scholars working in this latter vein are concerned with matters such as how populations classified as vulnerable by others (eg, by public health practitioners) are approached and imagined, as well as with how ‘vulnerability’ is itself constructed in practice (Grant 2016; Lupieri 2022; Montgomery 2012; Plotkin-Amrami and Brunner 2015). Some scholarship takes public health ascriptions of vulnerability almost for granted to strategically highlight and contest issues of epistemic or health-related injustice, while other work elides (rather than neatly separating) the empirical and analytical uses of vulnerability, as in the way medical ethics and bioethics foregrounds the term’s normative pull. In medical humanities and STS—as with elsewhere—vulnerability is a concept that can itself be explored, interrogated and critiqued, as well as a device to *enable* critique.

In this paper, we discuss the use of vulnerability to approach disease and disease risk, illustrating our insights using COVID-19 management protocols in the UK. Disease is, of course, not the only medical context in which ‘vulnerability’ is used, but it is an exemplary one. In the ongoing pandemic, both risk and vulnerability have been mobilised to describe public health decisions and private protective actions (Ahmad *et al*. 2020; De Togni *et al*. 2020; Flood, MacDonnell, and Philpott 2020; Moore 2020; Ten Have and Gordijn 2021). How societies approach future pandemics will be shaped by how both ‘disease risk’ and people, populations and indeed places deemed ‘vulnerable to disease’ are viewed. We contend that imagining certain groups as vulnerable necessarily involves particular conceptions of disease and how it moves in society, conceptions which can be related both to the somatic mechanisms of the disease and the ‘imagined biological’ (Pickersgill 2018). Accordingly, ascriptions of vulnerability not only raise questions about the politics of their production—they also necessitate and energise engagement about the politics embedded in concepts of disease themselves.

## Argument and outline

In what follows, we first offer a review of vulnerability within feminist and disability theory, setting out a fundamental philosophical tension in the concept. In brief, vulnerability is created vis-à-vis the social context and existing power distributions, and therefore could—and arguably should—be rendered otherwise. In other ways, though, vulnerability is a universal and unifying condition, the embracement of which could enable the affective and political recognition of the devalued aspects of us all.

Then, we discuss how both vulnerability and disease are commonly recruited by a range of social actors and institutions into a determinate, binary conceptualisation of normal versus abnormal. In this respect, health is posited as a normal state against which pathology is defined, and vulnerability is often similarly opposed to ‘resilience’—a quality increasingly expected, desired and required for living a ‘normal’ life. Yet, the boundaries between both resilience/vulnerability and health/disease are indeterminate. In part, this is because they evolve over time—as in the case of public health and medical metrics. It is also because they are processual and continually enacted: these dyads are not static states. We suggest that the tension inherent in the concept of vulnerability is, in fact, its strength, if used in a way that preserves indeterminacy. This is because of the concept’s potential to enable the recognition of inequities and their amelioration in non-deterministic ways, and without ‘othering’ certain groups or kinds of people on the basis of their capacities and diverse abilities. That is, vulnerability could be strategically deployed to move us beyond a normal/abnormal paradigm as we seek appropriate and beneficial ways of approaching disease and disease risk.

Next, we apply this insight by considering biomedical and public health conceptualisations of vulnerability. Using a few examples of the ambiguity and ambivalence shown towards vulnerability as it has been applied to COVID-19 in the UK, we illustrate how vulnerability is a key contemporary concept that is nonetheless put to ambiguous use. Building on this, we discuss several examples of attempts to codify and quantify vulnerability in order to address it, arguing that doing so makes it essentially replicate the function of ‘risk’ and eliminates much of what, in our view, makes vulnerability a valuable concept.

Finally, we speculate about what a non-deterministic application of vulnerability might look like, gesturing towards pluralistic ways of approaching disease (and pandemics in particular) that reflect contemporary social justice movements. Such movements require non-universalising and non-hierarchical approaches to problems and solutions. Crucially, people who might be classed *as* vulnerable need to be involved in the term’s application; it could best be used as a term of participatory engagement (Erikainen *et al*. 2021a). We suggest that pushing against the tendency to construe vulnerability in instrumental or technocratic terms, and instead keeping indeterminacy in play, would be beneficial in public health and policy spaces as well as more critical justice-oriented ones.

### Vulnerability and indeterminacy

Within our interdisciplinary group of STS-orientated scholars, we found that those more familiar with critical work on the concept of vulnerability were drawing largely from feminist and disability theory. Feminist and disability studies are mutually influencing, of course: both centrally understand vulnerability via intersectional power relations, and across both sets of literature there are fundamental philosophical tensions in the concept. Vulnerability is contingent yet universal, indicative of troubling power relations yet a feature of the human experience, valuable yet devalued by attempts to ameliorate it.

Vulnerability is ‘an especially fraught concept for feminist theory’ because of the ways it is gendered (Cunniff Gilson 2016: 94). Attributing vulnerability to specific groups—in this case, female or feminine groups—obscures how ubiquitous vulnerability is, while also stigmatising it. Frailty, incapacity, disability—and disease—are similarly stigmatised and opposed to the unmarked ‘norm’ despite being conditions all people encounter for some portion of their lives. The stigmatisation of weakness for people of any gender is related to this quality’s association with femininity and thus hierarchical subordination. Feminist scholarship has recognised that ‘vulnerability is predominantly understood as *feminising* and subsequently as negative, scary, shameful and, above all, something to be avoided and protected against’ (Dahl 2017). At the same time as wanting to protect *against* vulnerability, vulnerability implies the need for protection. It is an undesirable state that makes moral claims on those in positions of power, simultaneously evoking revulsion and sympathy.

Gendered power relations are, of course, conditioned by other axes of power including race, class, national origin, cis-gender, sexuality and (dis)ability. Vulnerability (and related concepts like dependence; Martin 2021) is associated with an ostensibly *ideal* state of appropriate or respectable white and middle-class femininity, which obscures how normative, respectable vulnerability is not available to (or necessarily desired by) racialised, poor or otherwise marginalised feminine subjects, nor to men, as hegemonic masculine positions are built through denying men’s vulnerability and localising it on the feminised (Butler, Gambetti, and Sabsay 2016). In transexclusionary politics, cis women who are generally white and middle-class claim a position of vulnerability to exclude others under a logic of protection and risk (Pearce, Erikainen, and Vincent 2020). Where it intersects with anticolonial critique, feminist theory interrogates global power relations and how feminist movements have been racialised and classed (eg, Vergès 2019). Critique of white western ‘saviour’ discourses wherein ‘vulnerable women’ from ‘elsewhere’ are victimised and presumed to need rescuing calls into question the linkage between vulnerability and lack of agency (Abu-Lughod 2002). It also overlaps with critical development studies and political economy analyses of the way geopolitical dependencies are created by those with power.

If power relations are the key *context* for feminist framings of vulnerability, the idea that vulnerability is relational—and therefore produced—is, for us, the key *theoretical* insight among them. Vulnerability is always an effect of specific social and historical relations (Butler, Gambetti, and Sabsay 2016). Anticolonial work has traced how power operates to *establish* the disenfranchised as ‘vulnerable populations’, and in so doing, render them unagentic, and consequently, subject to further control. This includes though discourses of protectionism (*ibid*). Indeed, as feminist political theorist Brown (2020) argues, the price of ‘protection’ of any sort is always some degree of dependence and unfreedom.

Within biomedical contexts, for instance, this risks an effacement of autonomy that might implicitly reify patriarchal and Western norms (Manda-Taylor *et al*. 2021). The idea of vulnerability as a constitutive condition of feminine embodiment is consequently challenged; instead, accounts describing its production are called for. Especially in feminist ethics of care and literatures on relational autonomy, vulnerability is reconceptualised via centring relations instead of the mythical independent subject of conventional moral theory (eg, Mackenzie 2014; McCrossin *et al*. 2022). Nussbaum (2006) and others have emphasised how human flourishing is dependent on social relations with others, including relations of care under conditions of vulnerability, challenging atomistic notions of autonomy.

Relational thinking is highly resonant with the social model of disability (SMD), which posits that society *makes* people disabled through its organisation around particular kinds of bodies. This means that many vulnerabilities often associated with disability are contingent rather than inherent, and tightly linked to the barriers and affordances given by our social environments and wider ecosocial systems (Filipe *et al*. 2021b; Scully 2013; Shakespeare 2017). With the rise of SMD in the 1980s and 1990s, vulnerability and frailty temporarily faded from disability studies, and issues of pain, desire and affect were also erased in ways that were complicit in the denial of embodied difference (Shildrick 2005), including by oversimplifying disability in a relativist way as *mere* difference (Hirschmann 2018). Since then, many disability studies scholars have embraced discussions of impairment, the body and vulnerability (eg, Price 2015; Slater 2014). And yet, it is precisely the desire to disavow vulnerability, not only in the context of disability but of all forms of embodiment, that can be regarded as generative of anxiety in the individual psyche and cultural imaginary.

Discussions about vulnerability in disability studies are marked by tension between ‘the phenomenological reality of living with impairment and the notion that vulnerability, like disability itself, is a socially constructed entity that shores up oppressive and limiting barriers in the lives of people with disabilities’ (Shildrick 2005: 557; see also Burghardt 2013). We join some disability theorists in wanting to keep this tension in play and embrace vulnerability’s complexities to articulate a more generous and honest appraisal of the human condition (Ginsburg and Rapp 2020). Shildrick suggests reconceptualising disability ‘in the light of an always already vulnerability as the condition not only of all bodies, but of all embodied selves’ Shildrick 2009. She situates present conceptions of disability as wholly enmeshed with ‘the relentless binaries of western epistemology that set health against illness, conformity against disparity, the perfect against the imperfect, the self against the other’, and asks what it would look like to address vulnerability alongside a critique of normative standards and their cultural specificity. Furthermore, she invites us to recognise that the binary structure itself is unstable. By delinking vulnerability from abnormality, we could approach vulnerability as something ubiquitous, if unevenly distributed: as ‘the risk of ontological uncertainty for all of us’ (p223; see also Fineman 2008).

Universalising vulnerability and emphasising the need to be with others are projects on which disability and feminist theory overlap, especially in queer studies and crip studies (Clare 2017; Piepzna-Samarasinha 2018). Embracing our mutual vulnerability as a shared human condition and being reminded that we all are constituted in and through relations to others can highlight our collective need for connection and care (Hewer 2019). Vulnerability can be seen as a positive link between ourselves and others, as the basis for ethical responsiveness and not merely a condition that we are obliged to ameliorate (Cunniff Gilson 2016). This approach to vulnerability critiques its conflation with victimisation and passivity, instead reimagining vulnerability as a condition of the very possibility of resistance (Butler, Gambetti, and Sabsay 2016), and thereby a valuable state. Like those in disability studies, feminist theorists have called for a new conceptualisation that moves beyond dualistic framings of powerful/weak, vulnerable/invulnerable and positive/negative, and instead develops more nuanced, non-reductive conceptions of vulnerability.

Yet while all are affected by ‘corporal vulnerability’ and life’s general precarity, some are disproportionately so (Butler 2004). Vulnerability is unequally distributed, and this gives rise to obligations to ameliorate and redress the social, cultural and environmental factors that exacerbate such inequality. Distributive justice ethicists have argued for this imperative across diverse realms; the ethical distribution of vulnerability would need to encompass the diversity of communities’ experiences as well as recognise the need for their engagement in policy and knowledge production (Erikainen *et al*. 2021a; Schlosberg 2004). Which manifestations of vulnerability result from and perpetuate existing distributions of power, reinforcing what is understood as normal and desirable, and which manifestations make space for the devalued and divergent aspects of us all? When vulnerability’s relational and universal aspects are considered together, we might understand it ‘as a dynamic and contextually heterogeneous aspect of the life of a ‘person-in-environment’… as sensitivity and openness to the living milieu and, thus, a harbinger of both negative and positive potentials’ (Filipe, Lloyd, and Larivée 2021a). Its indeterminacy of meaning enables a reframing that foregrounds relationality and universality while also permitting questions about distributive inequalities.

### Disease, resilience and normality

Like vulnerability, disease is a flexible category; indeed, the two are often synthesised together. Work from across a range of disciplines has shown that categories of disease, illness and disorder are not straightforwardly determined by that which we call ‘nature’. Rather, diseases are defined through social negotiations that reflect interested and situated perspectives (Dumit 2006; Jutel 2014; Rosenberg and Golden 1992). Indeed, ascriptions of vulnerability are part of what *makes* disease (Swallow 2020), reflecting the implicit idea that being vulnerable is opposed to being healthy and ‘normal’. What is considered normal is politically and culturally powerful, but also socially contingent and evolving.

We see both vulnerability and disease as being recruited, both popularly and professionally, into binary conceptualisations of normal versus abnormal. Thinking of pathologies as abnormal is an historically persistent, if also situated and evolving, way of approaching them within biomedicine (Canguilhem 1989), and disease states have long been defined in relation to their ‘opposite’, health (see Filipe, Lloyd, and Larivée 2021a). In many ways vulnerability is becoming similarly opposed to ‘resilience’, a quality increasingly required of (and seen as desirable by) citizens and subjects within a neoliberal political era (Bracke 2016; Evans and Reid 2014; Neocleous 2013). This emphasis on resilience exerts normative pressure; that is, one feels one *should* be resilient, and deviations from resilience become understood as problems. Consider the enthusiastic public response to Brené Brown’s 2010 TED talk and subsequent bestselling book (Brown 2012) encouraging personal vulnerability as the means to a fulfilling life, which reassured listeners that they did not, in fact, need to always be resilient but could embrace their vulnerabilities—ironically, ultimately in the service of stronger, healthier (and presumably more resilient) selves.

Attempts to ‘fix’ vulnerability—meaning, to pin it down and then remove it—are deeply entwined with binary thinking. But although resilience and vulnerability get recruited into a binary, they are both ambiguous and ambivalent terms. We have laid out vulnerability’s internal tensions above, and resilience has its own. As opposed to ‘security’ or ‘safety’, resilience implies injurability from which one should ‘recover’, or at least persevere through the damage, perhaps becoming stronger and better through the experience. The relationship between resilience and vulnerability, then, is also ambiguous. Are they actually ‘opposite’ in any sense? Does their dialectic of injury and recovery build towards greater strength, or greater weakness?

Resilience has been critiqued by scholars for individualising the burdens of the neoliberal abdication or reprioritisation of governmental responsibility. The concepts of *risk and security* formed the basis of an earlier era in Euro-American late capitalism, whereas *vulnerability and resilience* are becoming more relevant and resonant concepts as neoliberal trends towards individual responsibilisation and the fragmentation of political and governmental accountability intensify, amidst decreasing ‘safety nets’ (Harvey 2007; Rose 2007). Vulnerability and resilience also become more salient as we collectively face daunting and diffuse challenges like climate change, chemical toxicity and zoonotic pandemics—engaging imaginaries of permanent threat (Neocleous 2013). They are hallmarks of an era when being secure or safe seems to be the outlier rather than the norm. With his concept of ‘reflexive modernity’, Beck (1992) foresaw that modernity (at least, Euro-American versions of it) will involve increasing individualisation and the requirement of constructing one’s own biography as if freely chosen, but within structural constraints (including the pressure to self-construct in the first place). In the way it is often used in popular and policy settings, resilience becomes an expectation of individual people, cities, islands and so on in the absence of efforts at making environments that are conducive to flourishing, from workplace economics to the planetary climate. Everyone struggles with the heaped-up demands of resilience, even if these are differentially distributed.

If resilience moves theory away from the possibility of safety, which should be recognised as a myth in the context of current political and environmental challenges (Ford 2020; Shotwell 2016), it should not be used to simply excuse easily preventable endangerment. This echoes how some vulnerability is innate and universal, and some created by inequitable structures, policies and histories—while the differences and distinctions between these might be hard, if not impossible, to straightforwardly demarcate, not least given their interpolations. Both vulnerability and resilience are worth cultivating*,* a concept that encompasses both inherency and intentionality. Neither resilience nor vulnerability are quintessentially *bad;* rather, harm happens when resilience becomes an expectation and when vulnerability becomes unacceptable.

Harms also happen when either quality is seen as a property of individual entities instead of something produced in and through relationships. The individuation of vulnerability and resilience follows the way that risk is likewise individuated under neoliberalism, and further blurs the distinction between vulnerability and risk in public health. Identifying individuals and groups as ‘vulnerable’ is in effect a way of identifying them as ‘at risk’ without actually (or at least, necessarily) having to resort to the technicalities of demonstrating risk, and to consequently invite resilience instead of risk mitigation. The indeterminacy within vulnerability can, then, be used to equivocate between ungrounded perceptions of insecurity and the kinds of interventions that are more normally triggered by the calculus of risk. We argue that this is not the optimal use of the concept.

### Applying vulnerability

Moving away from binary thinking would enable harms and flourishing to be distributed in diverse and flexible ways. However, efforts to address inequalities by designing policies and interventions around ‘vulnerability’ often involve codifying a version of vulnerability and thereby attempting to smooth over tensions, contradictions and differences that, we argue, are useful to keep in play. Indeed, ambiguities often creep in anyway. Discourse around COVID-19 in the UK—where all of the authors are currently based—is a timely example of how vulnerability can produce ambiguity in applied settings. Such ambiguity is distinct from the concept’s inherent indeterminacy, which could lead instead to multiple, changeable and specific applications that are not characterised by ambiguity.

Public health governance in the UK is a devolved matter, resulting in four different sets of COVID-19 guidelines within England, Scotland, Wales and Northern Ireland. Much work was undertaken to harmonise the approach across the four health systems, including advice from the UK Joint Committee on Vaccination and Immunization (JCVI). However, decisions about public health measures were not homogenous across the UK. Government COVID-19 protocols within the four nations have featured questions about whether 'vulnerable groups' (who may or may not have been designated such before the pandemic) should be prioritised for access to vaccinations and recommendations that people with specific health conditions should ‘shield’—meaning take extra precautions and avoid potentially infectious activities. Such people were designated ‘clinically extremely vulnerable’ (CEV). This language implied and sometimes required clear definitions of who is vulnerable. However, deciding who to include—and indeed, whether to use the term at all—is not straightforward. It was wildly controversial and an opaque process that happened at varying degrees throughout the pandemic; indeed, specific health conditions were added and removed from the clinical definition of CEV throughout the pandemic, with minimal consultation from—or communication with—patients (Ryan 2021).

A key National Health Service (NHS) webpage was titled ‘Who is at high risk from coronavirus (clinically extremely vulnerable)’ in 2021; yet, in 2022, the NHS reported that ‘People are no longer being called clinically extremely vulnerable’ (NHS 2022a) and that guidance exists for people *previously* considered CEV (DHSC 2022; see also ScotGov 2022). Following this, the guidance simply specifies ‘people at high risk from coronavirus’ or people at ‘highest risk’. The category of ‘clinically extremely vulnerable’ was linked to a specific policy intervention, namely the initial advice on ‘shielding’ directed to those deemed to be CEV, and in this regard, the term’s later discontinuation was evidently linked to the decision in September 2021 to drop the advice on shielding in England and to end the highest risk list (formerly CEV) in May 2022 in Scotland (decisions informed by widespread access to vaccines). This strongly suggests that ‘clinically extremely vulnerable’ only existed so long as there was a policy that was targeted at that group, and that the designation of this special category was precisely about identifying a class of people who need exceptional attention because they are not ‘normal’. Indeed, a later version of the page tells those previously advised to shield that now ‘you can follow the same advice as everyone else’ (NHS 2022b).

On the website as originally published, the most striking thing is the profound ambiguity regarding who is seen to be ‘vulnerable’, a group at once explicitly equated with and implicitly understood to be in some ways different from those deemed ‘at risk’. Equating vulnerability with risk serves to anchor it to a well-established medical empirical calculus, including the identification of specific risk factors—in this instance, those factors being primarily other medical conditions. By contrast, the shift from risk to vulnerability is accompanied by moves that loosen and potentially break that anchoring. In particular, the original website invokes medical judgement on whether a given individual needs to shield, freeing doctors to override the risk-based evaluation and decide for themselves who should be given what kinds of advice. At the same time, and conversely, the shift from risk to vulnerability elides a range of other observable and known risk factors for disease including race and class, which are passed over in silence. These are not, incidentally, insignificant: people living in the poorest Scottish neighbourhoods were twice as likely to die as their wealthier neighbours; the death rate was 86.5 per 100 000 in the poorest fifth of Scottish neighbourhoods, compared with 38.2 in the richest fifth (Gordon 2020).

Classifying certain groups as vulnerable not only normalises but perpetuates existing distributions of power (Ganguli-Mitra 2020); in other words, labelling some people as in need of special protection can mask the conditions that disempower them in the first place. Talk of ‘extreme vulnerability’ and ‘shielding’ designates a group who warrant special protection, consideration and treatment, and yet the policy shifts responsibility for care back onto those designated vulnerable. All the website did (speaking for the NHS and Department of Health and Social Care) is advise those who doctors judge to be ‘CEV’ on steps that *they,* the CEV themselves, should take if possible and if their employers permit (see Ryan 2020). Seen like this, vulnerability does not confer any rights or privileges; it is merely the basis for an appeal to the goodwill of those with power, meanwhile engendering sudden and significant disruption to one’s life at short notice. Being part of this category did confer early access to vaccination, for those who wished it and whose particular vulnerabilities allowed them to take it up, although also raised concerns about these groups being ‘guinea pigs’ for a rapidly produced vaccine. In this respect, the shift to vulnerability, as represented in this policy website, is not just ambiguous: it is also profoundly *ambivalent*, at once invoking and abdicating responsibility. This ambivalence might extend to public health more generally, and the dilemmas it currently faces in how to exercise its limited powers of intervention in the public domain, given the shift in the 1990s to ‘lifestyle’ and individual ‘responsibilisation’ (Petersen and Lupton 1996; Young *et al*. 2019).

Designating a category of people that should ‘shield’ while doing little to enable this shielding or proactively protect those so categorised is also inherently ambivalent about what vulnerability means and requires. In the first 20 months of the pandemic, CEV was used in a particular way, establishing how certain conditions put one at increased risk of ill-health/death from COVID-19. Within this, there was ambiguity about vulnerability to existing illness versus vulnerability to COVID-19; for example, CEV included people who were immunosuppressed (vulnerability to infection), those on dialysis (vulnerability from existing illness), as well as the very elderly (age-related vulnerability) (Ganguli-Mitra *et al*. 2020). Although the UK and Scottish governments used the same terms (based on JCVI definitions/agreements), their deployment of ‘vulnerable’ in policy—particularly in providing support to those deemed vulnerable—was not the same. For instance, working with local councils, the Scottish Government offered additional protections for those shielding, including specific, although temporary, labour rights (eg, extended absence from work and policies of working from home), as well as objects and services to support self-care (eg, drug, food, and care delivery and telephone support for the isolated). In Scotland, letters from the chief medical officer (CMO) to people who were CEV counted as ‘sick notes’ and could be used to claim sick leave (the CMO regularly wrote to everyone on this list with updates). However, this ran into significant issues when that sick leave was used up, and the practice was discontinued when the highest risk list ended in May 2022. In England, support such as food delivery for those shielding was significantly restricted and highly criticised. Services for those deemed ‘vulnerable’ were heavily policed, indicating significant material differences in access to and experiences of formal government support (see Ryan 2020a). Government support was supplemented in both countries by local groups, councils and organisations, but the differences underscore our point that ambiguity and ambivalence characterise the application of ‘vulnerability’ in this context.

Although measures to proactively ensure the well-being of those designated ‘vulnerable’ were rather thin, people construed as existing within that category have been used to justify the implementation of relatively restrictive public health measures, while indeed being subject to enhanced limits on their *own* mobility and social practices. The need to shield raises the question of risk from community transmission; is risk therefore from the community itself? In polarised public and social media discussions, restrictive measures were alternately embraced as acts of solidarity and demonised as impositions on individual freedoms. In debates over issues from mask-wearing to vaccination to ‘herd immunity’, rhetoric about ‘the vulnerable’ appeared to evoke both sympathy and contempt—which can carry with them their own overlapping politics of abjection and exclusion. More nuanced perspectives, which rendered problematic the criminalisation of interpersonal care while recognising and supporting the necessity of many public health responses, were rather less apparent. Such nuance, we suggest, could result from engaging with vulnerability’s indeterminacy instead of treating it as basically equivalent to risk.

### From codification to indeterminacy

Attempts to codify vulnerability in COVID-19 protocols resonate with medical (and, in some cases, social) models of risk that are evident in some broader public health and bioethics applications of vulnerability. For example, Bourgois *et al*. (2017) propose a scale of ‘structural vulnerability’ for use in clinical practice as a way of measuring factors external to the individual patient, ideally then used in physician advisory panels for community interventions. Hurst (2008) advocates a definition of vulnerability that would systematise kinds of ‘wrongs’ people might incur and determine their likelihood in ‘identifiable increments’. Luna (2019) advises determining specific ‘layers’ of vulnerability to nuance it as a ‘label’. Others distinguish ‘biological vulnerabilities’ (eg, pre-existing diagnoses) from social and economic vulnerabilities, glossing over the entanglement of the biological and the social. For some, ‘how we treat our vulnerable’ is a premise of ‘just society’ discourses that aim to address inequalities; however, these play into the dynamics of privilege and protection discussed above, whereby those who are protected are rendered unagentic and disabled.

Although some scholars argue that codifying and quantifying inequalities is essential to changing them (Braveman and Gruskin 2003; Krieger 1999, Patrick 2005), and that structural problems require structural changes (Krieger 2021), we see a different value in avoiding prescriptive or universalising analyses. As opposed to calculating risk, allowing vulnerability to remain indeterminate would in fact open doors to more effective (because variable) application that would lend itself to distributing power for decision making and determining what ‘help’ and protection is needed among affected groups, who will have various answers to these questions. We recognise the clear bureaucratic logic for codifying a concept such as vulnerability; that is, codification makes it actionable as a tool of management and governmentality. Yet working out how to build variability into bureaucratic systems is, we suggest, key to making them ethical and responsive, and we conclude with thoughts on how to do so. It is an opportunity for not only a shift in perspective but a shift in power dynamics, in line with calls for epistemic justice (Almassi 2018; Chung 2021; Fricker 2013). In their article responding to a *Lancet* editorial on COVID-19 and vulnerability, Ahmad *et al*. (2020) take issue with the Editors’ opening question, ‘What does it mean to be vulnerable?’, arguing that ‘The lived experiences of vulnerable groups are defined by a form of epistemic injustice—the dismissal of the knowledge of their own lives and needs’. The question should not be answered on their behalf.

Medicalised framings of disability/vulnerability primarily focus on bodily function, autonomy and life-quality measurements and, thereby, obfuscate the sociohistorical, environmental and institutional underpinnings of health, well-being and ability that are crucial for addressing future pandemics in just ways (Pickersgill, Manda-Taylor, and Niño-Machado 2022; Ten Have and Gordijn 2021), as well as non-ableist perspectives on human diversity and interdependency (Filipe *et al*. 2021b). If calculations of risk/vulnerability do consider social determinants of health (ie, the social conditions in which people are born, grow, live, work and age; Braveman and Gottlieb 2014), they do not often do so in ways that vary among communities and are determined and prioritised by those communities. While the social determinants of health model’s encoded concern for remedying inequality is admirable, it relies on ‘determinacy’ and thus sets us on a path towards prescriptive solutions. A merely additive view of what needs to be included to assess vulnerability does not go far enough in addressing injustice. It ‘fixes’ vulnerability, in the sense of holding it in place, even while also trying to remove it. Repairing injustice requires flexibility, variability, nuance and changed power dynamics.

When vulnerability essentially takes the place of risk as an (ostensibly) universally measurable thing, well-intentioned efforts can reproduce the inequalities they seek to remedy. This includes through externally imposed categories and ideas of what healthy bodies and practices look like, which may uphold and even exacerbate conditions of inequality by not accounting for local conditions, priorities and ways of making meaning (Yates-Doerr 2020). Determining ‘upstream’ causes of illness versus treating the ‘downstream’ effects can be helpful, but does not go far enough in changing healthcare systems and environments. By contrast, undertaking interventions in conversation with those impacted, aligning external expertise with existing community-based expertise, and treating both health and health interventions as relational processes could be transformative of inequalities and their attendant health risks. Along the lines of the ‘situated view’ represented by the SMD above, the burden need not be on individuals to change when social changes could render their ‘inability’ less relevant, or not a hindrance at all. As inherently relational, vulnerability could be a powerful concept towards this end.

Social and environmental inequalities produce vulnerabilities (including to ‘natural’ disasters; Kelman 2020). These inequalities are structural, built into hierarchies that are global in reach if not homogenous, along lines of class, gender, race, and via the echoes and present-day manifestations of colonialism. Yet naming those disadvantaged by these hierarchies is both complicated and problematic, since it is at least partially through labelling that people are made vulnerable, in need of protection and silenced. Some argue that risk is a preferable term because labelling at-risk communities ‘vulnerable’ is a process of otherising and essentialising that also erases these communities’ resilience (Marino and Faas 2020). Other theorists take this one step further and point out that emphasising class and racial inequalities in the distribution of harm can re-entrench those inequalities by stigmatising groups or kinds of people as ‘damaged’ (Murphy 2017). As an indeterminate concept, vulnerability, we argue, could meaningfully orient critical attention to inequity while avoiding reifying that inequity, if it were used to enable communities and groups to define their own needs and priorities on an ongoing basis. Vulnerability could be a starting point for solidarity and mutuality that *precludes* deterministically and prescriptively advancing a limited view of what is desirable, as long as vulnerability is understood as inherently flexible and plural.

Operationalising this is, certainly, complicated. We acknowledge we are raising challenging questions to which we too struggle to find the answers, and that translating our intellectual position into the strong relational logics of existing systems, which are organised around the need to simplify ambiguity, is necessary if we are to go beyond wishful thinking. Scholarly work has identified intersecting, complex needs presented by pandemic vulnerabilities, which could serve as a starting point. The need to slow disease transmission must be weighed against, for example, the time-sensitive need to access abortion (Joffe and Schroeder 2021), which is already more challenging for marginalised communities and intersected with the increase of domestic abuse during lockdown. In some ways, as a response to considerable distress the pandemic restrictions catalysed sociotechnical innovation among groups long regarded as vulnerable (including people seeking abortions, as access to at-home medical abortions increased in some places; Reynolds-Wright *et al*. 2022). The normalisation of home working positively affected some chronic pain and fatigue sufferers (Evans *et al*. 2021), yet was a ‘double-edged sword’ for others with disabilities (Barden *et al*. 2023) and has ongoing and complicated repercussions for those with care responsibilities, not to mention the ways that risks facing ‘key workers’ unable to work from home have been eclipsed. In the face of intensely heteronormative emphasis on ‘home’ and nuclear family ‘bubbles’, some LGBTQIA communities reworked how to practice bodily intimacy and communality, conducting ethically complex ‘experiments with mortality’ that questioned the future-oriented social priorities represented in biomedical recommendations, while working out how to communicate and negotiate this with others (Lim 2020).

Scholarly work has also revealed how governmental and bureaucratic systems operating from the logic of reducing ambiguity did not successfully distribute COVID-19 care across populations. Existing inequalities were not only correlated with increased disease risk, but these inequalities interacted with wider psychosocial and policy responses to COVID-19, thus deepening as part of pandemic management (Pickersgill 2020). Racial differences in how state and institutional power functions became stark within the context of supposedly universally applied policies; as Rouse (2021) writes in the context of the USA, ‘whites in May protested against state biopolitics; Blacks protested against state necropolitics’. Protests against coercive interventions in the general population may be viewed alongside the amplification of disability-specific lawful violence (Spivakovsky and Steele 2022). And marginalised groups’ differential access to vaccines not only exacerbated their risk of getting sick, but was often demonised in popular, policy, and some scholarly circles as ‘vaccine hesitancy’ instead of structural failure, rhetoric that deepened prejudices around religious and ethnic minorities in the UK (Kasstan 2021).

These insights are starting points for conversations and participatory experiments in change-making, to explore alternative, implementable models of bureaucratic and governmental relations. Our centre has done work imagining and interrogating public and patient involvement, particularly in the digital era (Erikainen *et al*. 2019), and through this line of inquiry has identified tools that might be useful in operationalising vulnerability in participatory, flexible, diverse and non-hierarchical ways. For example, in a report commissioned for the National Endowment for Science, Technology and the Arts on ‘Moving Beyond Conventional Engagement Methods’, some of us evaluated ‘mini publics’ like citizen’s juries, community-based monitoring and oversight programmes, e-democracy and crowdsourcing methods, workshops and dialogue, and arts-based and game play methods (Erikainen *et al*. 2021a). Once models for variable policy making and administration are imagined, they can be tested, and gradually implemented. Such engagements, and subsequent openness to implementing the ideas generated, will be essential for an ongoing, non-deterministic and justice-oriented application of vulnerability in disease contexts. The more familiar such modes of interaction are within governments and public health professions, the more readily they could be adapted and deployed in future disease and pandemic situations. Better still, establishing channels of engagement whereby at-risk and structurally marginalised communities are already being consulted about ways of mitigating and embracing their vulnerabilities would allow for appropriate infrastructure and policies to be established *before* a disease event. This can and should be done in conjunction with more traditional qualitative research identifying specific changes that would be beneficial for particular groups (eg, Coyle and Atkinson 2019). The intersecting, complex needs discussed above require a concept of vulnerability that foregrounds power sharing, flexibility and multiple ‘norms’.

## Conclusion

Vulnerability and disease both get recruited into an unhelpful binary conceptualisation of normal versus abnormal. We argue that emphasising indeterminacy in how we enact these categories could be analytically helpful, potentially liberating and even transformative of injustices in ways that resonate with contemporary justice movements—particularly for epistemic justice that entails the redistribution of decision-making power. Key to this is accepting and engaging the idea that vulnerability cannot and should not be determined externally, and embracing its indeterminacy—tensions, plurality, polysemy—instead of attempting to ‘fix’ it. Vulnerability is, and should remain, a more capacious term than—for example—risk, and should be used differently. It is a more morally charged concept that lends itself to bigger debates about (un)fairness and (relational) autonomy.

In pluralising the practical meanings of vulnerability—in particular, what it requires, and from whom—and sharing the process of determining these things with those who will be affected, vulnerability could become destigmatised. Through this process, space could be opened up for the many ways that all people, at some point in their lives, are unwell, experience impairment, and have physical or psychological capacities that do not neatly align with tasks essential to them in particular social milieu or which they are made responsible for (Ginsburg and Rapp 2020), while still retaining analytical and political focus on people and communities who are even more explicitly *made* vulnerable through the actions of more powerful actors and institutions. Vulnerability could allow for the recognition of inequality as undesirable and socially stratified while not attempting to ‘solve’ it in deterministic ways. Rather than becoming fixed into labels, unidirectional causalities, or top-down universalising metrics, vulnerability could be used to insist on relational, context-specific understandings of disease risk, its situated meanings and helpful measures.

Thinking with vulnerability as a multivalent and capacious term that encompasses not only our shared weaknesses but shared strengths could lead to thinking analogously about health and disease, reaching outside the normal/abnormal paradigm. In suggesting this, we are positioned alongside disability justice activists (eg, Abrams and Orsini 2022; Clare 2017; Piepzna-Samarasinha 2018). Given that both vulnerability and resilience are worth cultivating*,* what would it look like to view health and disease with generosity instead of erasure? We are, of course, not suggesting that disease itself should be cultivated—but that recognising it as ubiquitous instead of abnormal could lead to better ways of mitigating the harms associated with it. As some have argued for cultivating ‘a good death’ in the face of the inevitable (Rushing 2021), what would it look like to cultivate ‘a good vulnerability’ or ‘a good experience of disease risk’ in the face of equally inevitable experiences of illness and other forms of disease—even as we might challenge social, political and economic structures that worsen these? We need only to look at the experiences of isolated older adults in residential care during the COVID-19 pandemic to see that encounters with disease and with attempts to forestall or address it are complex, and normatively and affectively multivalent (van der Geugten, Jacobs, and Goossensen 2022). What kinds of suffering and harm are rendered unimportant in a medicalised culture in which managing viral transmission out of existence is the only seriously considered response (Lim 2020)?

Vulnerability, as an indeterminate and relational concept, could help us envision systems of structures and values that pluralise notions of the ‘bad’ and enable ways to minimise it, while also refusing to make it the basis of difference and exclusion. It would include recognition of structural vulnerabilities and oppose ‘responsibilizing’ what is beyond individual control, while avoiding the limiting view that structural vulnerabilities are primarily barriers to individual autonomy. The violence of structural vulnerabilities could be kept in tension with the positive value of shared vulnerability and mutual interdependence. This tension is analogous to the tension across different versions of feminism over whether to fight for inclusion in that which has been valued (from which women have been excluded), versus reclaiming and embracing what has been devalued (which risks fixing women’s association with it). A similar tension exists in disability studies over whether to disavow frailty and become as able as possible, versus embrace the nuanced embodied experience of having variable capacities and their particular joys, desires and sorrows. All such tensions rely on and perpetuate binary thinking, which is not surprising as binaries are incredibly durable and hard to think beyond. Indeed, even in articulating this point we find ourselves rehearsing that which we critique. Indeterminacy is difficult because it refuses to permit a reliance on familiar binaries as shortcuts. This is the potential—and the challenge—of vulnerability.

## Data Availability

Data sharing is not applicable as no data sets were generated and/or analysed for this study.
